# PSMB9 Promotes the Malignant Progression of Colorectal Cancer by Regulating the PI3K/Akt Pathway

**DOI:** 10.32604/or.2026.082652

**Published:** 2026-08-13

**Authors:** Wen Gao, Xingyu Zheng, Yanan Hu, Rui Zou, Yongan Zhou, Xiang Song

**Affiliations:** 1 The Second Clinical Medical College of Shanxi Medical University, Taiyuan, China; 2 Department of Oncology, the Second Hospital of Shanxi Medical University, Taiyuan, China; 3 The Sixth Clinical Medical College of Shanxi Medical University, Taiyuan, China; 4 Department of Blood Transfusion, the Second Hospital of Shanxi Medical University, Taiyuan, China

**Keywords:** Colorectal cancer, proteasome 20S subunit beta 9 (PSMB9), PI3K/Akt pathway, immunoproteasome, tumor invasion

## Abstract

**Background:** As a core component of the immunoproteasome, the β1i subunit (proteasome 20S subunit beta 9, PSMB9) is involved in antigen processing and presentation and regulates anti-tumor immune responses. PSMB9 is aberrantly overexpressed in colorectal cancer. However, the precise mechanisms through which PSMB9 contributes to the initiation, progression, and immune regulation of colorectal cancer remain unclear. This study aims to investigate the expression characteristics and biological functions of PSMB9 in colorectal cancer, and to further elucidate the molecular pathways underlying its role in colorectal cancer initiation and progression. The findings are expected to provide a theoretical basis for the development of targeted therapeutic strategies against colorectal cancer. **Methods:** PSMB9 expression in colorectal cancer was analyzed using the TCGA, GEO, GEPIA, and HPA databases, and further validated in normal colonic epithelial cells and colorectal cancer cell lines by RT-qPCR and Western blot. Lentiviral transduction was used to establish stably transduced HCT116 and SW480 cell lines with PSMB9 knockdown and overexpression, respectively. Cell proliferation, migration, and invasion were assessed by CCK-8, colony formation, wound healing, and Transwell assays. Key proteins of the PI3K/Akt signaling pathway were detected by Western blot. **Results:** PSMB9 was significantly upregulated at both the mRNA and protein levels in colorectal cancer tissues and cell lines. PSMB9 knockdown significantly inhibited the proliferation, migration, and invasion of colorectal cancer cells, while overexpression enhanced these malignant phenotypes. Mechanistically, PSMB9 exerted oncogenic effects through activation of the PI3K/Akt signaling pathway. **Conclusions:** PSMB9 promotes the malignant progression of colorectal cancer by regulating the PI3K/Akt signaling pathway.

## 1 Introduction

Colorectal cancer (CRC) ranks as the third most commonly diagnosed malignancy worldwide and the second leading cause of cancer-related mortality, imposing a substantial burden on global healthcare systems [1]. Currently, the clinical treatment of CRC primarily relies on conventional therapeutic strategies, including surgical resection, chemotherapy, and targeted therapy [2,3]. In recent years, immunotherapy has emerged as a pivotal frontier in oncology research, with substantial advances achieved in the development of immunotherapeutic strategies for CRC. Nevertheless, the clinical application of such immunotherapies continues to face significant obstacles, including immune-related adverse events, limited therapeutic efficacy, and acquired drug resistance [4,5,6]. For patients with advanced or metastatic CRC, clinical outcomes remain dismal, with a 5-year overall survival rate of only 14% [7]. Therefore, there is an urgent need to identify novel therapeutic targets, elucidate their underlying signaling mechanisms, and develop innovative therapeutic approaches to further prolong survival and improve the quality of life of CRC patients.

The proteasome 20S subunit beta 9 (PSMB9) gene is located within the major histocompatibility complex (MHC) region on human chromosome 6 and encodes the catalytic subunit β1i of the immunoproteasome. Its expression is predominantly induced by interferon-gamma (IFN-γ) [8]. As a core component of the immunoproteasome, PSMB9 functions as a critical mediator in antigen processing during immune responses [9]. Upon malignant transformation, PSMB9 participates in the degradation of oncogenic proteins to generate optimal antigenic peptides, which efficiently activate CD8^+^ T cells and trigger anti-tumor cellular immunity [10]. In addition to its antigen-processing function, PSMB9 is involved in protein degradation, the maintenance of cellular homeostasis, and the regulation of cellular physiological functions [11,12]. Mounting evidence has demonstrated that PSMB9 is aberrantly upregulated in various malignant tumors, including CRC, cervical squamous cell carcinoma, cholangiocarcinoma, esophageal cancer, and clear cell renal cell carcinoma, suggesting that its role in tumorigenesis is complex and multifaceted [13]. In melanoma, high PSMB9 expression exerts an inhibitory effect on tumor progression [14,15]. In breast cancer, elevated PSMB9 expression is associated with a better prognosis in triple-negative breast cancer [16]. In muscle-invasive bladder cancer, patients with high PSMB9 expression exhibit a trend toward prolonged overall and progression-free survival [17]. Conversely, in low-grade gliomas (LGGs), patients with high PSMB9 expression display shorter survival and a worse prognosis [18]. However, the specific molecular mechanism by which PSMB9 modulates CRC progression and immune regulation remains largely unclear.

In this study, we first integrated data from multiple public databases. Through bioinformatics analyses, we systematically investigated the differential expression of PSMB9 in CRC, as well as the enrichment patterns of PSMB9-associated genes and the enrichment characteristics of corresponding signaling pathways. Based on these analyses, we further explored the regulatory role of PSMB9 in the proliferation, migration, and invasion of CRC cells, and elucidated the core molecular mechanisms underlying its functional effects. Ultimately, this study aims to characterize the expression pattern of PSMB9 in CRC, clarify its regulatory effects on the malignant phenotypes of CRC cells, and illuminate the underlying molecular mechanisms, thereby identifying PSMB9 as a promising therapeutic target for CRC.

## 2 Materials and Methods

### 2.1 Acquisition of Data from Public Databases

Colon adenocarcinoma RNA-seq data were downloaded from the Colon Adenocarcinoma (COAD) project of The Cancer Genome Atlas (TCGA, https://portal.gdc.cancer.gov/) via the Genomic Data Commons portal, comprising 483 primary tumors and 41 matched normal colon samples. A microarray dataset (GSE39582) containing 566 colon adenocarcinomas and 19 normal colon tissues was obtained from the Gene Expression Omnibus (GEO, https://www.ncbi.nlm.nih.gov/geo/). For external validation, we performed PSMB9 expression comparisons using the Gene Expression Profiling Interactive Analysis (GEPIA, http://gepia.cancer-pku.cn/) platform, which contains a quality-controlled subset of 275 TCGA-COAD tumor samples and 349 normal colon tissues from the Genotype-Tissue Expression (GTEx) project. PSMB9 protein expression in normal colon and tumor specimens was examined using immunohistochemistry images from the Human Protein Atlas (HPA, https://www.proteinatlas.org/).

### 2.2 Immune Infiltration Analysis

CIBERSORT was used to perform deconvolution analysis of the relative abundances of 22 immune cell subsets in CRC tissues and matched normal controls [19]. Subsequently, correlation tests were applied to evaluate the associations between PSMB9 expression levels and the infiltration levels of each immune cell type. To visually present the analytical results, scatter plots, correlation heatmaps, and chord diagrams were generated using the R packages ggplot2 (version 3.5.1), corrplot (version 0.92), and circlize (version 0.4.16), respectively.

### 2.3 Gene Enrichment and Pathway Analysis

To investigate the potential biological functions of PSMB9 in CRC, we calculated Pearson correlation coefficients between PSMB9 expression and that of all other genes based on RNA-seq data from the TCGA-COAD cohort. Genes with a correlation coefficient greater than 0.5 and a *p* value less than 0.05 were defined as significantly correlated with PSMB9. Subsequently, the official human gene symbols of these genes were submitted to the Database for Annotation, Visualization, and Integrated Discovery (DAVID) online tool (version 6.8) for Gene Ontology (GO) functional annotation and Kyoto Encyclopedia of Genes and Genomes (KEGG) pathway enrichment analysis. A false discovery rate (FDR) less than 0.05 was used as the significance threshold to screen for significantly enriched GO terms and KEGG pathways. The significant GO terms and KEGG pathways were ranked by FDR values in ascending order, and the top six entries from each were selected as the core results.

### 2.4 Cell Culture and Lentiviral Transduction

We acquired the human CRC cell lines HCT116 (T1027) from Applied Biological Materials Inc. (Zhenjiang, Jiangsu, China) and SW480 (C5233) from Baidi Biotechnology Co., Ltd. (Hangzhou, Zhejiang, China). The normal colonic epithelial line NCM460 (C5710) was also obtained from Baidi Biotechnology Co., Ltd. HCT116 and SW480 cells were cultured in high-glucose Dulbecco’s Modified Eagle Medium (DMEM) (Baidi Biotechnology, ST002-500), while NCM460 cells were cultured in DMEM/F12 (Baidi Biotechnology, L104-500). All complete media were supplemented with 10% fetal bovine serum (FBS) and 1% penicillin-streptomycin. Cultures were maintained at 37°C in a humidified incubator with 5% CO_2_ (Thermo Fisher Scientific, model 311, Beijing, China).

Lentiviral particles for PSMB9 knockdown and overexpression were obtained from Applied Biological Materials Inc. The knockdown vector (pLenti-U6-shRNA-CMV-GFP-2A-Puro) encoded a short hairpin RNA (shRNA) targeting PSMB9 under the U6 promoter (shRNA insert sequence: 5′-GCTGCAAATGTGGTGAGAAATTTCAAGAGAATTTCTCACCACATTTGCAGC-3′) and co-expressed GFP and a puromycin resistance gene from the CMV promoter. The overexpression vector (pLenti-GIII-CMV-PSMB9-C-term-3xFlag-CBH-GFP-2A-Puro) carried the full-length PSMB9 coding sequence with a C-terminal 3xFlag tag driven by the CMV promoter, while GFP and puromycin resistance were co-expressed under the CBH promoter. HCT116 and SW480 cells in the logarithmic growth phase were seeded into 6-well plates (Baidi Biotechnology Co., Ltd., H803000) at a density of 5 × 10^4^ cells per well and cultured for 24 h. Upon reaching 50%–60% confluence, the culture medium was replaced with fresh complete medium containing 8 μg/mL polybrene (Servicebio, G1803, Wuhan, China), and lentiviral particles were added at a multiplicity of infection (MOI) of 10. At 72 h post-infection, the cells were selected with complete medium supplemented with 2 μg/mL puromycin (Servicebio, G4017) for 7 consecutive days. Knockdown and overexpression efficiencies were confirmed by measuring PSMB9 mRNA and protein levels using reverse transcription quantitative real-time PCR (RT-qPCR) and Western blot. Only cell populations that achieved a knockdown efficiency of ≥70% or an overexpression fold change of ≥2 were harvested for subsequent experiments.

LY294002 (Y243556), a highly selective PI3K inhibitor, was purchased from Beyotime Biotechnology (Shanghai, China). After standard preparation and dilution according to the manufacturer’s instructions, the inhibitor was used at a final concentration of 10 μM. PSMB9-overexpressing HCT116 and SW480 CRC cells were treated with LY294002 for 24 h, after which cell samples were collected for subsequent Western blot analysis. All experiments included three biological replicates and were repeated independently three times.

### 2.5 RT-qPCR

Cells in the logarithmic growth phase were harvested and pelleted by centrifugation. Total RNA was extracted using TRIzol^TM^ reagent (Thermo Fisher Scientific, 15596026CN). RNA concentration was determined using a spectrophotometer (Thermo Fisher Scientific, 840-317500), and the A260/A280 ratios for all samples were confirmed to be within the range of 1.8–2.0 to ensure RNA purity. Complementary DNA (cDNA) was synthesized using the All-In-One 5X RT MasterMix kit (Applied Biological Materials Inc., G592), which includes a genomic DNA removal step. The reverse transcription mixture was prepared on ice according to the manufacturer’s instructions: 4 μL of All-In-One 5X RT MasterMix, 1 μg of total RNA, and nuclease-free water to a final volume of 20 μL. The mixture was incubated at 37°C for 15 min, followed by 60°C for 10 min and 95°C for 3 min. The resulting cDNA was diluted 10-fold with nuclease-free water and used as the template for quantitative real-time PCR (qPCR). Each 20 μL qPCR reaction contained 10 μL of BlasTaq^TM^ 2X qPCR MasterMix (Applied Biological Materials Inc., G891), 0.5 μL each of forward and reverse primers, 2 μL of diluted cDNA, and 7 μL of nuclease-free water. The thermal cycling conditions were as follows: initial denaturation at 95°C for 3 min, 40 cycles of 95°C for 15 s and 60°C for 1 min. GAPDH served as the internal reference gene, and the relative expression level of PSMB9 mRNA was quantified using the 2^−^^ΔΔCt^ method. All samples were tested in triplicate, and three independent experimental replicates were performed. The primer sequences are listed below:

PSMB9-F: 5′-GGAGCTCCATGGGATAGAACTG-3′

PSMB9-R: 5′-TGTGCAGACAAGTCCTCTCG-3′

GAPDH-F: 5′-GAAAGCCTGCCGGTGACTAA-3′

GAPDH-R: 5′-GCCCAATACGACCAAATCAGAG-3′

### 2.6 Western Blot

Following harvesting, cells from each transfection group were lysed on ice for 30 min in pre-chilled RIPA lysis buffer (Servicebio, G2002) supplemented with protease (Servicebio, G2008) and phosphatase (Servicebio, G2007) inhibitors at a volumetric ratio of 100:1:1 (RIPA:protease inhibitor:phosphatase inhibitor). After centrifugation at 12,000× *g* for 20 min at 4°C, the supernatants were collected as total protein extracts. Protein concentrations were determined using a bicinchoninic acid (BCA) kit (Servicebio, G2026). Protein samples (20 μg per lane) were resolved by electrophoresis at a constant voltage of 160 V. The separated proteins were transferred onto a polyvinylidene fluoride (PVDF) membrane using a wet transfer system at a constant current of 300 mA for 30 min. After transfer, the membrane was blocked with protein-free rapid blocking buffer (Servicebio, G2052) for 10 min at room temperature and then incubated overnight at 4°C with the following primary antibodies (all sourced from HUABIO, Hangzhou, Zhejiang, China): anti-PSMB9 (1:500, ET7107-24), anti-PI3K (1:1000, ER64588), anti-p-PI3K (1:1000, HA721672), anti-Akt (1:2000, HA721870), anti-p-Akt (1:2000, ET1607-73), and anti-GAPDH (1:50,000, ET1601-4). After three washes with Tris-buffered saline containing Tween 20 (TBST), the membrane was incubated with horseradish peroxidase (HRP)-conjugated goat anti-rabbit IgG (1:50,000) at room temperature for 1 h, followed by three additional washes with TBST. Target bands were visualized using an enhanced chemiluminescence (ECL) reagent (Servicebio, G2020) and imaged using a chemiluminescence imaging system (Servicebio, SCG-W5000 PLUS). Band intensities were quantified using ImageJ software (version 1.53, National Institutes of Health, Bethesda, MD, USA). All experiments were independently repeated at least three times to ensure reproducibility.

### 2.7 CCK-8 Assay

The cell suspension was adjusted to a concentration of 2 × 10^4^ cells/mL, and 100 μL was seeded per well into a 96-well plate (Baidi Biotechnology Co., Ltd., H803006). The plate was placed in a humidified 37°C incubator with 5% CO_2_ to allow cell attachment and growth. After complete cell attachment, 10 μL of CCK-8 reagent (Servicebio, G4103) was added to the wells designated for each time point (0, 24, 48, and 72 h post-attachment). At each time point, the plate was incubated at 37°C in the dark for 2 h, and the absorbance at 450 nm was measured using a microplate reader (Thermo Fisher Scientific, 51119080). Wells containing culture medium alone (without cells) were included as blank controls. The absorbance value of the blank control was subtracted from that of each test well, and the corrected absorbance values were used to reflect cell proliferation activity. Three independent biological experiments were performed, each with triplicate technical replicates per condition.

### 2.8 Colony Formation Assay

Stably transfected HCT116 and SW480 CRC cells were seeded into 6-well plates at a density of 500 cells per well. The plates were gently swirled to ensure even distribution and then placed in the incubator. During incubation, the complete medium was replaced every 3–4 days. After 14 days of culture, the culture was terminated, and the wells were gently washed twice with PBS. One milliliter of 4% paraformaldehyde solution (Beyotime Biotechnology, P0099) was added to each well, and the cells were fixed at room temperature for 30 min. Then, 1 mL of crystal violet (Servicebio, G1014) staining solution was added to each well for staining at room temperature for 20 min. After staining, the wells were rinsed gently with deionized water until the background was clear and no excess dye remained. The plates were inverted and air-dried at room temperature. Finally, Whole-well images of each well were captured under a uniform light source. Colonies were counted using ImageJ software (version 1.53, National Institutes of Health, USA). Each condition was tested in three biological replicates, each run in triplicate.

### 2.9 Wound Healing Assay

Five parallel lines, spaced 0.5 cm apart, were drawn on the bottom of a 6-well plate to serve as reference marks for subsequent image localization. Cells were seeded into 6-well plates and cultured overnight to form a confluent monolayer. Once 90%–100% confluence was reached, a linear scratch was created perpendicular to the reference lines using a 200 μL pipette tip at a constant speed. After scratching, the wells were gently washed three times with PBS to remove detached cells, and the medium was replaced with serum-free DMEM (Baidi Biotechnology Co., Ltd., ST002-500) for further culture. Scratch closure was observed and photographed under an inverted microscope (Olympus CKX41, Tokyo, Japan) at 0, 24, and 48 h post-scratch. The scratch area at each time point was measured, and the cell migration rate was calculated using the following formula: Migration rate (%) = (initial scratch area—scratch area at a given time point)/initial scratch area × 100%. All experiments were independently repeated at least three times to ensure reproducibility.

### 2.10 Transwell Assay

Matrigel (Baidi Biotechnology Co., Ltd., 3D200-010) was diluted 1:8 with pre-chilled serum-free DMEM (Baidi Biotechnology Co., Ltd., ST002-500) on ice and mixed thoroughly. A volume of 60 μL of the diluted Matrigel solution was added to the upper chamber of a Transwell insert (Corning, USA). The insert was then incubated at 37°C for 2 h to allow the Matrigel to solidify and form an artificial basement membrane. Subsequently, 200 μL of cell suspension at a density of 1 × 10^5^ cells/mL was added to the upper chamber, and 600 μL of DMEM containing 10% FBS was added to the lower chamber as a chemoattractant. For each group, three replicate wells were set up, and the culture plate was incubated in a CO_2_ incubator at 37°C for 48 h. After incubation, the inserts were fixed and then stained. Following staining, the inserts were rinsed repeatedly with PBS, and the upper surface of the membrane was gently wiped with a wet cotton swab to remove non-migrated cells. The inserts were allowed to air-dry upside down, after which multiple fields of view were randomly selected under an inverted microscope (Olympus CKX41, Tokyo, Japan) and images were captured. Migrated cells on the lower surface of the membrane were counted using ImageJ software (version 1.53, National Institutes of Health), and statistical analysis was performed. To ensure the stability and reliability of the research conclusions, the experiment was independently repeated at least three times.

### 2.11 Statistical Analysis

Data analysis and visualization were performed using GraphPad Prism 9.0 (GraphPad Software, San Diego, CA, USA) and R version 4.4.1 (R Foundation for Statistical Computing, Vienna, Austria). For normally distributed measurement data, comparisons between two groups were performed using the independent samples *t*-test, while comparisons among multiple groups were performed using one-way analysis of variance (ANOVA). When the data did not follow a normal distribution, non-parametric tests were applied. For correlation analysis, based on the actual distribution characteristics of the data, the Pearson correlation method was selected for normally distributed data, and Spearman’s rank correlation method was used for non-normally distributed data. A *p* value < 0.05 was considered statistically significant.

## 3 Results

### 3.1 PSMB9 Expression in CRC

**Figure 1:**
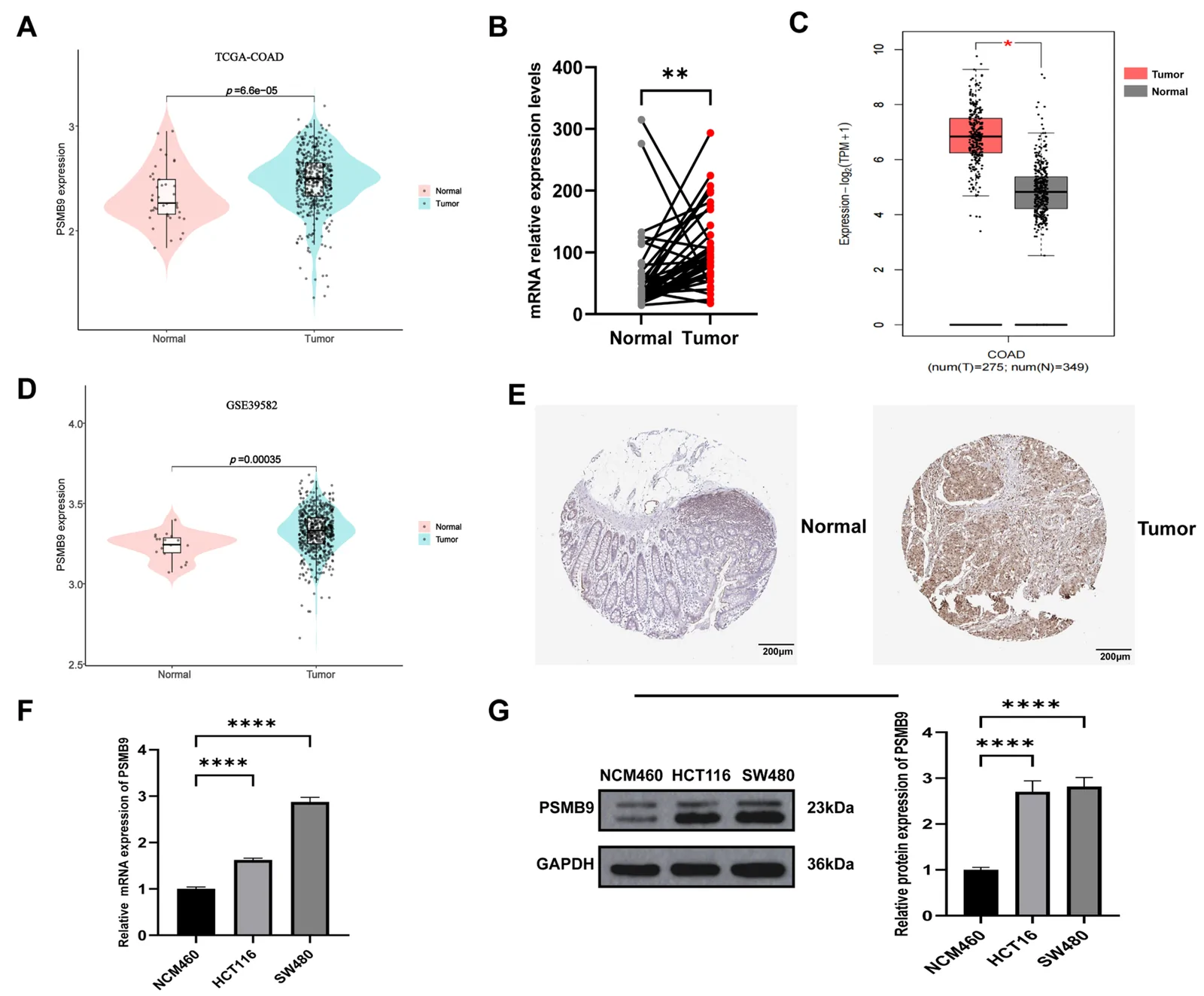
**Proteasome 20S subunit beta 9 (PSMB9) is overexpressed in colorectal cancer (CRC).** (**A**) Violin plot of PSMB9 expression in CRC tissues and normal colorectal tissues based on the TCGA-COAD database. (**B**) Relative mRNA expression levels of PSMB9 in 41 pairs of matched CRC tissues and adjacent normal tissues from the TCGA-COAD dataset, based on RNA-seq data. (**C**) Box plot of PSMB9 expression in CRC tissues and normal colorectal tissues based on the GEPIA database. (**D**) Violin plot of PSMB9 expression in CRC tissues and normal colorectal tissues from the GSE39582 dataset. (**E**) Representative immunohistochemical images of PSMB9 protein expression in normal colorectal mucosa and CRC tissues based on the HPA database. (**F**) RT-qPCR assay for PSMB9 mRNA expression levels in normal colorectal mucosal cells (NCM460) and CRC cell lines (HCT116, SW480). (**G**) Western blot assay for PSMB9 protein expression levels in NCM460, HCT116, and SW480 cells. *p < 0.05, **p < 0.01, ****p < 0.0001.

Analyses of multiple datasets, including TCGA-COAD, GEPIA, and GSE39582, consistently revealed that PSMB9 expression was significantly upregulated in CRC tissues compared with normal tissues (Fig. 1A–D). Immunohistochemistry data from the HPA further confirmed elevated PSMB9 protein levels in CRC tissues relative to normal colorectal mucosa (Fig. 1E). At the cellular level, both RT-qPCR and Western blot analyses demonstrated markedly higher PSMB9 mRNA and protein expression in the CRC cell lines HCT116 and SW480 than in the normal colorectal mucosal cell line NCM460 (Fig. 1F,G). Collectively, these findings suggest that aberrant overexpression of PSMB9 may be associated with the malignant phenotype of CRC cells.

### 3.2 Functional Enrichment Analysis of PSMB9

**Figure 2:**
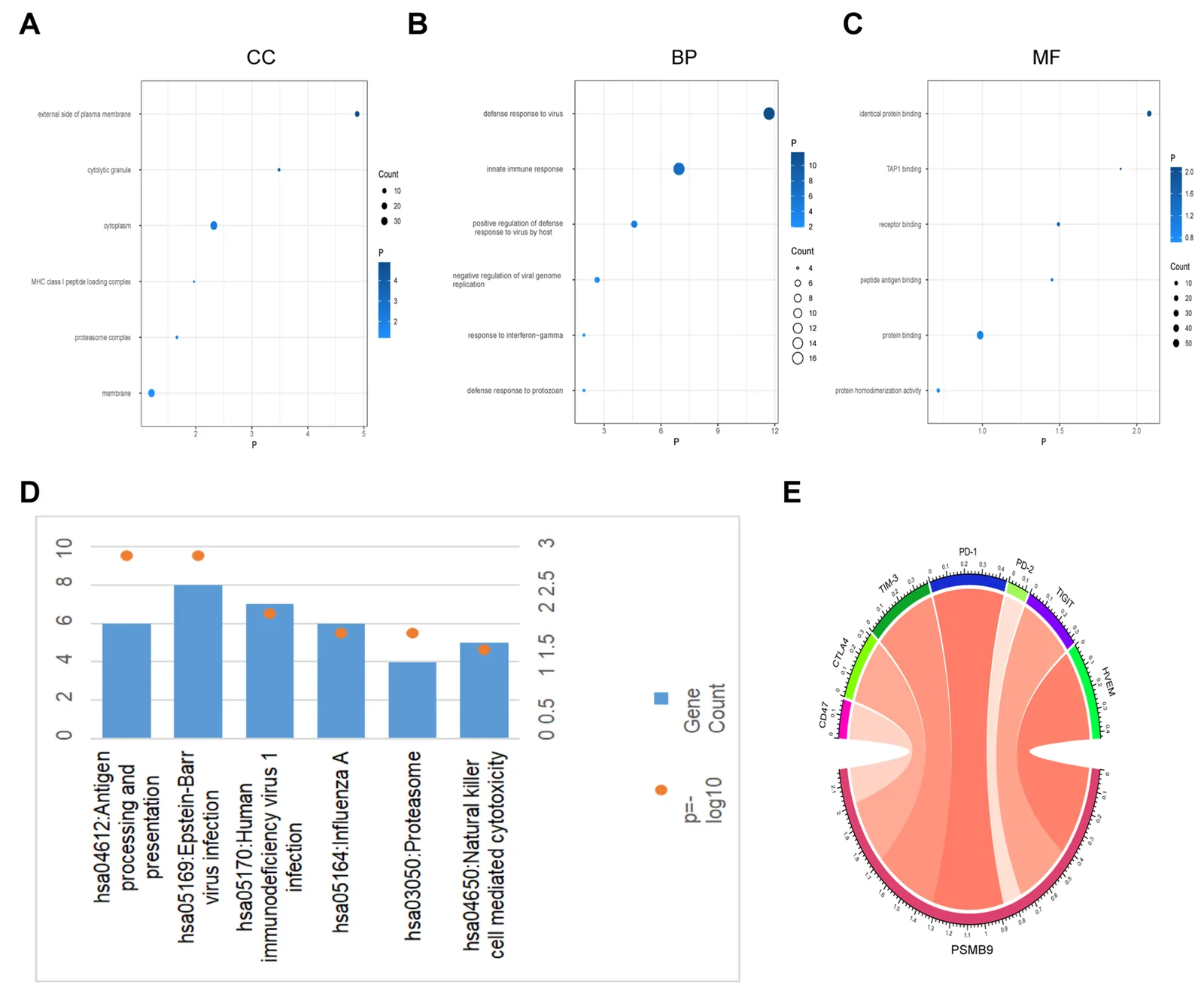
**Functional enrichment analysis of PSMB9.** (**A**) Cellular component. (**B**) Biological process. (**C**) Molecular function. (**D**) KEGG pathway enrichment analysis. (**E**) Pearson’s correlation between PSMB9 and inhibitory immune checkpoints.

GO functional enrichment analysis was conducted to characterize the biological roles of PSMB9. At the cellular component (CC) level, PSMB9 was primarily localized to the cytoplasm (Fig. 2A). At the biological process (BP) level, PSMB9 played a significant role in key immune regulatory processes, including the defense response to viruses, innate immune response, interferon-**γ**-mediated biological processes, and T cell activation (Fig. 2B). At the molecular function (MF) level, the primary function of PSMB9 was identified as specific interaction with transporter associated with antigen processing (TAP) proteins (Fig. 2C). Additionally, KEGG pathway enrichment analysis showed that the most significantly enriched pathway associated with PSMB9 was the antigen processing and presentation pathway (Fig. 2D). Pearson correlation analysis further revealed that PSMB9 expression was significantly positively correlated with several key inhibitory immune checkpoint molecules, including herpesvirus entry mediator (HVEM), TIM-3, PD-1, PD-L2, T cell immunoreceptor with immunoglobulin and immunoreceptor tyrosine-based inhibitory motif (ITIM) domains (TIGIT), and CD47 (Fig. 2E). Cumulatively, these results indicate that PSMB9 may regulate the tumor immune microenvironment by synergizing with inhibitory immune checkpoint molecules, thereby providing valuable insights for future investigations into its potential application in CRC immunotherapy.

### 3.3 PSMB9 Correlates with Immune Cell Infiltration in CRC

**Figure 3:**
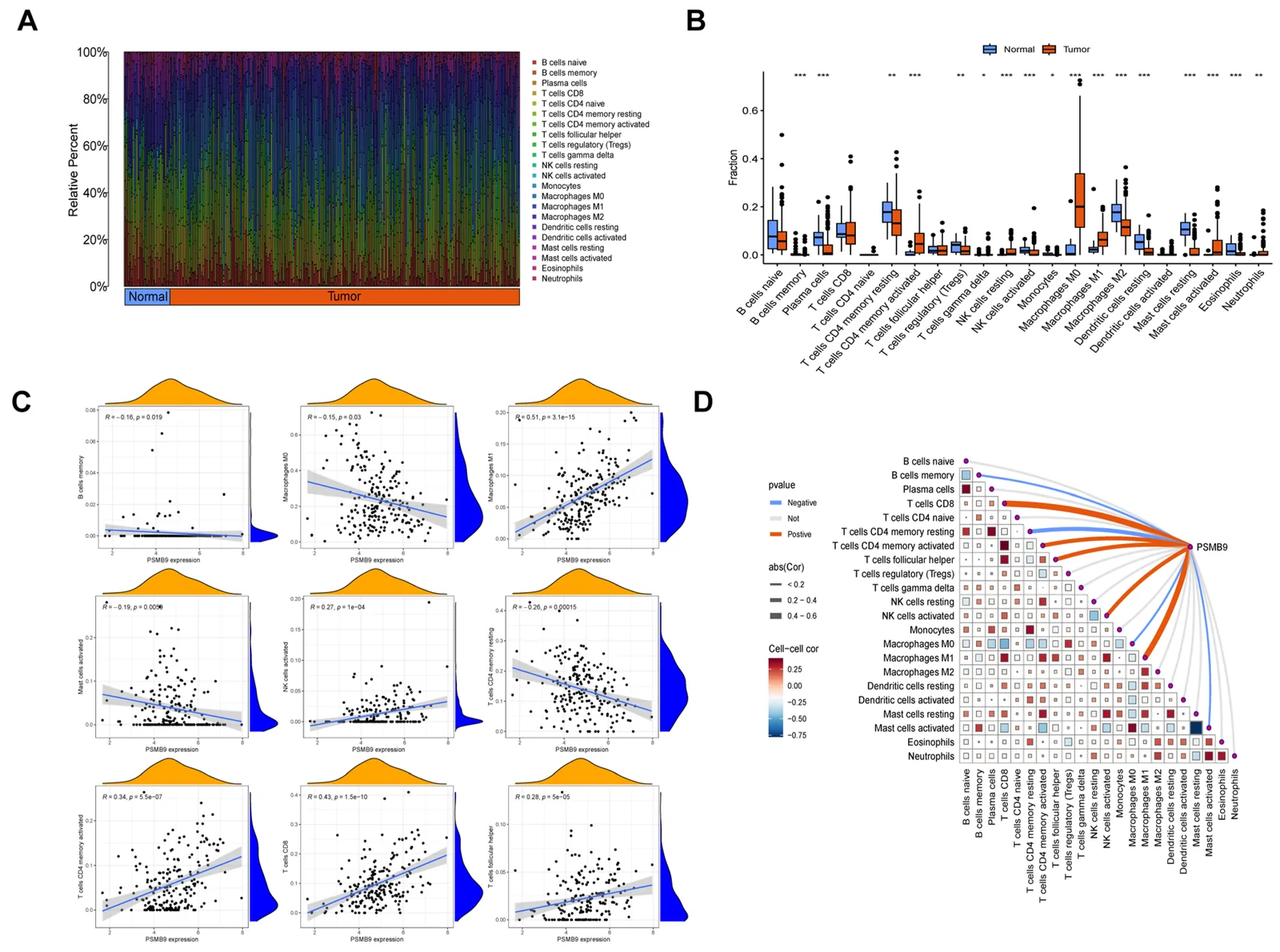
**Profiling of immune cell infiltration and its association with PSMB9 expression in CRC.** (**A**) Stacked bar plot showing the relative proportions of 22 immune cell subsets in normal and tumor samples. (**B**) Box plots comparing immune cell fractions between normal and tumor tissues. (**C**) Scatter plots showing correlations between PSMB9 expression and infiltration of representative immune cell subsets. (**D**) Correlation network illustrating associations between PSMB9 expression and all 22 immune cell subsets. *p < 0.05, **p < 0.01, ***p < 0.001.

Deconvolution analysis of the relative abundances of immune cell subsets in CRC tissues and adjacent normal tissues showed that, compared with normal controls, the proportions of T cells CD4 memory activated, macrophages M0 and macrophages M1 were significantly increased in tumor tissues (Fig. 3A,B). Further correlation analysis revealed that the PSMB9 expression level was negatively correlated with macrophages M0 and mast cells activated, whereas it was significantly positively correlated with M1 macrophages and T cells CD8 (Fig. 3C,D). These results reveal marked differences in immune cell infiltration between CRC and normal tissues and demonstrate that PSMB9 expression is closely associated with key immune cell subsets in the tumor microenvironment, suggesting that PSMB9 may actively shape and remodel the immune microenvironment of CRC.

### 3.4 PSMB9 Promotes the Proliferation of CRC Cells

**Figure 4:**
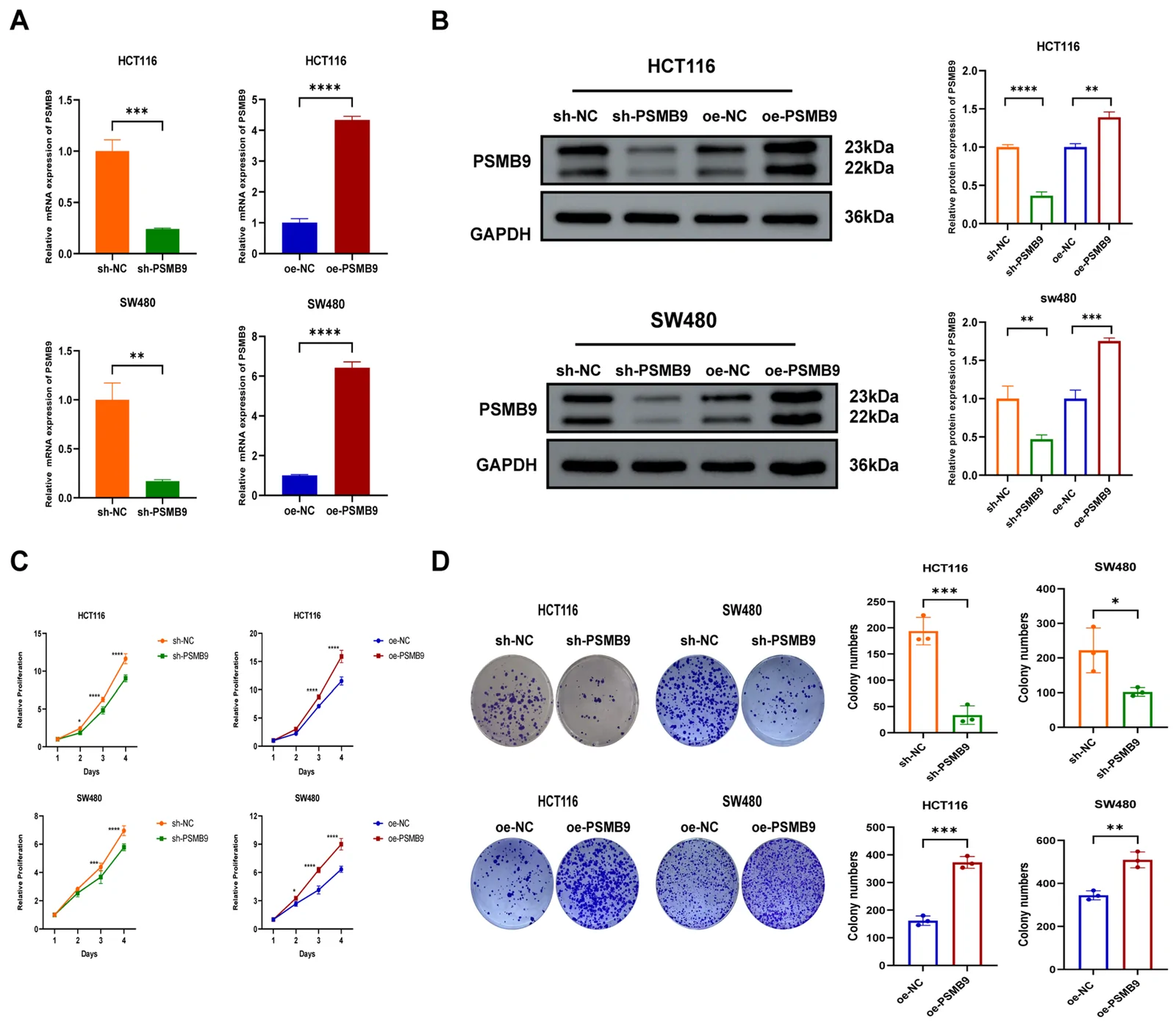
**Effect of PSMB9 on CRC cell proliferation.** (**A**,**B**) Validation of PSMB9 knockdown and overexpression efficiency. (A) Relative mRNA expression levels of PSMB9 determined by RT-qPCR. (**B**) Protein expression levels of PSMB9 examined by Western blot. The two isoforms, PSMB9-L (23 kDa) and PSMB9-S (22 kDa), are labeled. (**C**) CCK-8 proliferation assays in HCT116 and SW480 cells. (**D**) Colony formation assays. *p < 0.05, **p < 0.01, ***p < 0.001, ****p < 0.0001.

To further explore the biological functions of PSMB9 in CRC, the CRC cell lines HCT116 and SW480 were selected as research models. We generated stable PSMB9-knockdown (sh-PSMB9) and PSMB9-overexpressing (oe-PSMB9) cells, along with their respective negative controls (sh-NC, oe-NC). Knockdown and overexpression efficiencies were confirmed at both mRNA and protein levels by RT-qPCR and Western blot (Fig. 4A,B).

Cell proliferation capacity was evaluated using the CCK-8 assay. The results showed that sh-PSMB9 significantly reduced the proliferative activity of CRC cells, whereas oe-PSMB9 markedly enhanced cell proliferation (Fig. 4C). This trend was further validated by colony formation assays: sh-PSMB9 cells formed significantly fewer colonies, while oe-PSMB9 cells showed a prominent increase in colony number relative to controls (Fig. 4D). Together, these results demonstrate that PSMB9 promotes CRC cell proliferation.

### 3.5 PSMB9 Promotes the Migration and Invasion of CRC Cells

**Figure 5:**
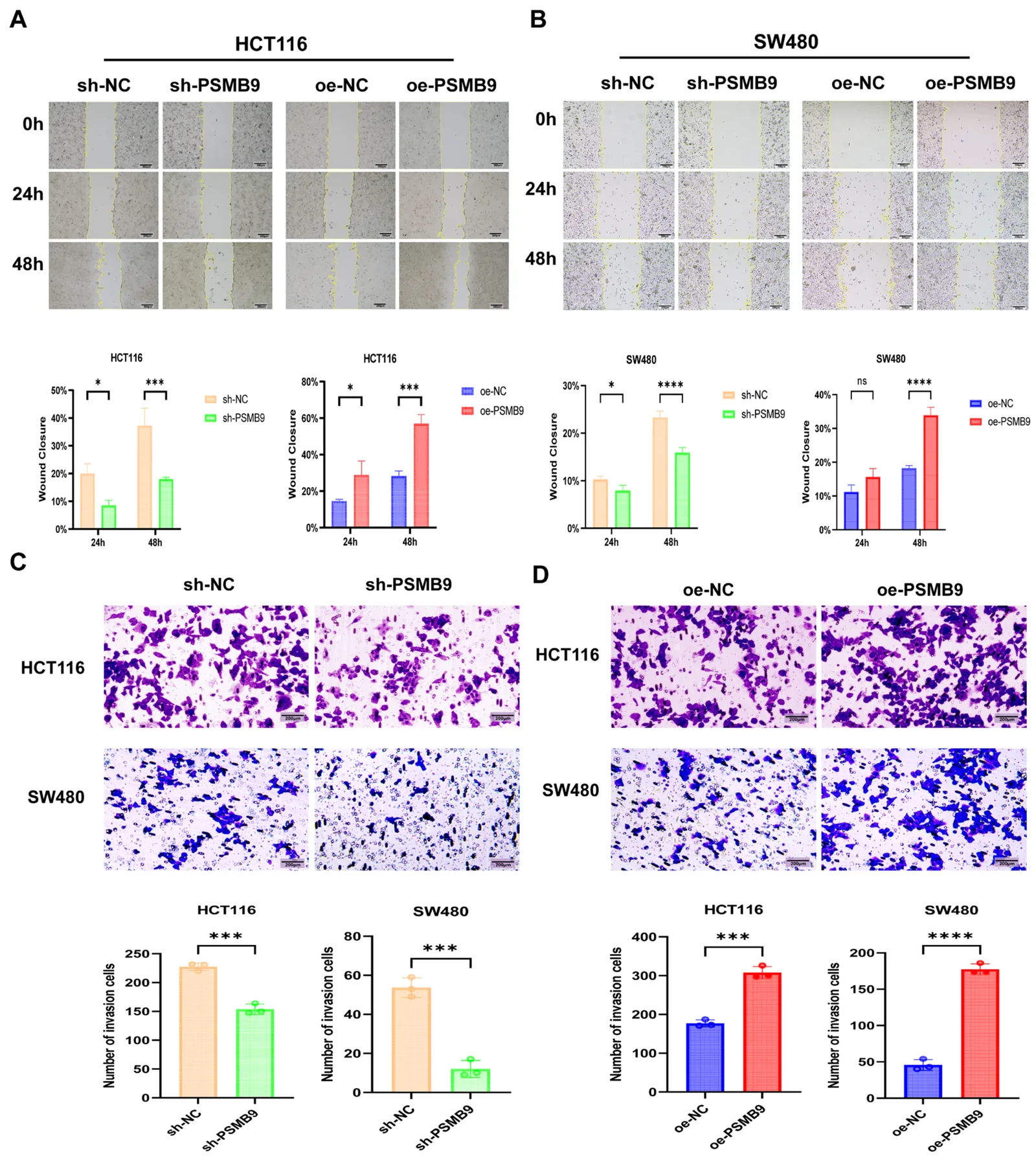
**Effect of PSMB9 on CRC cell migration and invasion.** (**A**,**B**) Wound healing assays showing the migratory capacity of (**A**) HCT116 and (**B**) SW480 cells with PSMB9 knockdown or overexpression. (**C**,**D**) Transwell invasion assays evaluating the invasive ability of (**C**) HCT116 and (**D**) SW480 cells with PSMB9 knockdown or overexpression. *p < 0.05, ***p < 0.001, ****p < 0.0001, ns no significance.

The effect of PSMB9 on migration and invasion was evaluated by wound healing and Transwell invasion assays. Wound healing assays revealed that oe-PSMB9 markedly increased the wound closure rate, whereas sh-PSMB9 significantly delayed the wound healing process (Fig. 5A,B). In the Transwell invasion assay, the number of cells that penetrated the Matrigel was significantly reduced in the sh-PSMB9 group compared with the control groups, while the number of invasive cells was notably elevated in the oe-PSMB9 group (Fig. 5C,D). Taken together, these findings consistently indicate that PSMB9 effectively promotes the migration and invasion of CRC cells *in vitro*.

### 3.6 Effect of PSMB9 on the Expression of PI3K/Akt Pathway Proteins in CRC Cells

**Figure 6:**
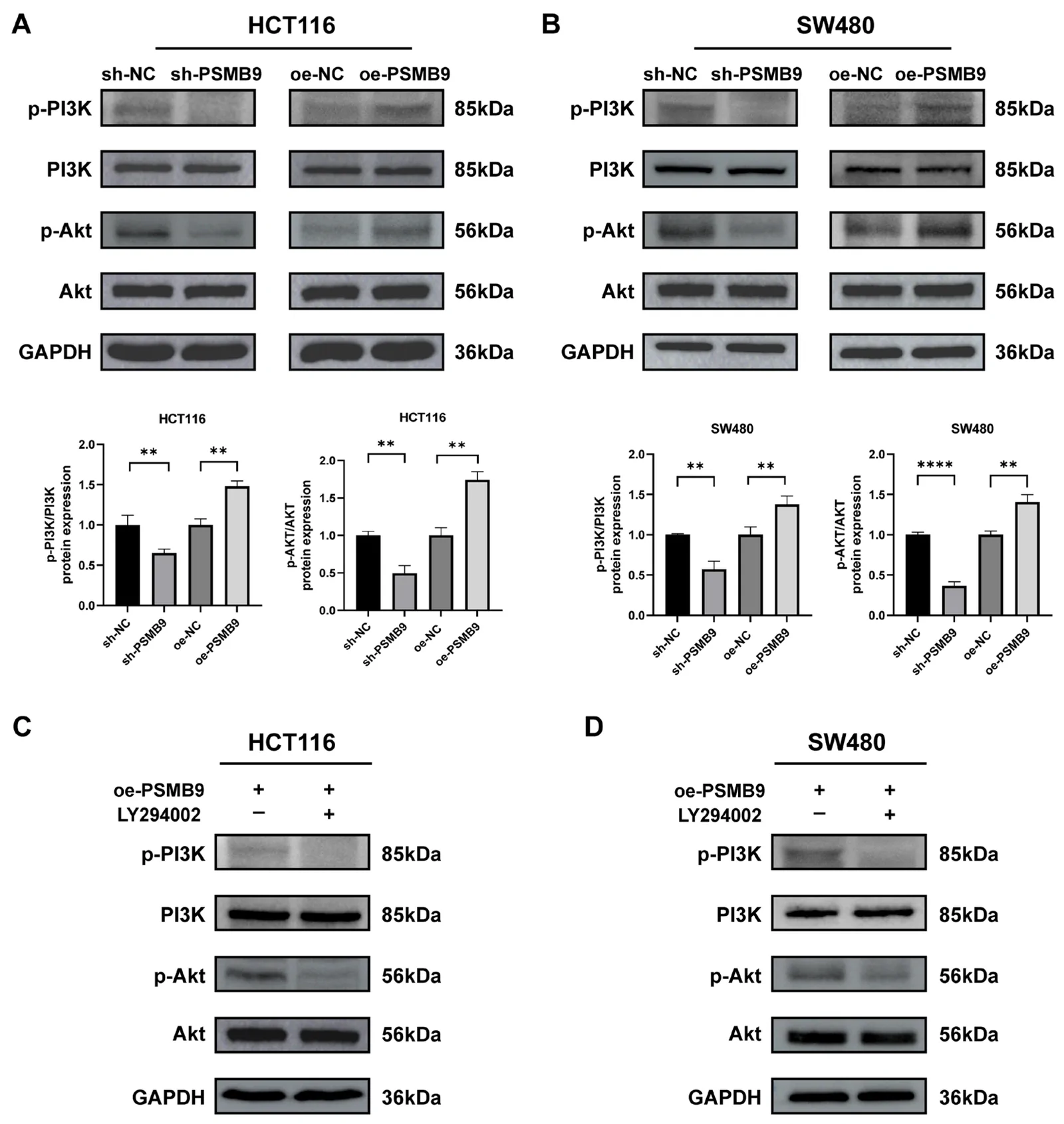
**PSMB9 regulates the expression of key proteins in the PI3K/Akt signaling pathway.** (**A**) Western blot analysis of p-PI3K, total PI3K, p-Akt, and total Akt in HCT116 cells with PSMB9 knockdown or overexpression. (**B**) The same assay was performed in SW480 cells. (**C**) HCT116 cells overexpressing PSMB9 were treated with or without the PI3K inhibitor LY294002, and the phosphorylation of PI3K and Akt was assessed by Western blot. (**D**) The same treatment and analysis as in (**C**) were applied to SW480 cells. **p < 0.01, ****p < 0.0001.

To elucidate the regulatory effect of PSMB9 on the PI3K/Akt signaling pathway, we assessed the levels of key pathway components by Western blot (Fig. 6A,B). PSMB9 knockdown (sh-PSMB9) significantly decreased the protein expression levels of phosphorylated PI3K (p-PI3K) and phosphorylated Akt (p-Akt) compared with the negative control (sh-NC). Conversely, PSMB9 overexpression (oe-PSMB9) markedly increased p-PI3K and p-Akt levels relative to the overexpression control (oe-NC). Notably, the total protein levels of PI3K and Akt remained unchanged across all groups.

To determine whether PSMB9-mediated activation of the PI3K/Akt pathway depends on PI3K activity, we treated PSMB9-overexpressing HCT116 and SW480 cells with the PI3K-specific inhibitor LY294002. Western blot analysis showed that PSMB9 overexpression upregulated p-PI3K and p-Akt levels, and this effect was largely abolished by LY294002 treatment (Fig. 6C,D). Taken together, these results indicate that PSMB9 activates the PI3K/Akt signaling pathway by promoting the phosphorylation of PI3K and Akt in CRC cells.

## 4 Discussion

Our study revealed that PSMB9 is highly upregulated in CRC relative to adjacent normal tissues, and its elevated expression is tightly correlated with indicators of malignant progression. Functional experiments showed that enforced expression of PSMB9 significantly enhances the proliferative, migratory, and invasive capacities of CRC cells, underscoring its involvement in tumor development. Mechanistically, we found that the pro-tumorigenic activity of PSMB9 is tightly coupled to activation of the PI3K/Akt cascade. Overexpression of PSMB9 markedly increased the phosphorylation of both PI3K and Akt, whereas knockdown of PSMB9 dampened their activation. Together, these findings not only shed new light on the molecular pathogenesis of CRC but also point to promising targets for therapeutic intervention.

PSMB9 is a key catalytic subunit of the immunoproteasome and, together with PSMB8 and PSMB10, forms its essential catalytic core [17,20]. By participating in antigen processing and presentation, it plays a crucial role in the immune response [21,22]. Within tumor cells, cytosolic proteasomes and immunoproteasomes degrade endogenous proteins into short peptides, which are then translocated into the endoplasmic reticulum lumen by TAP transporters and bind to newly synthesized MHC class I molecules to form peptide-MHC complexes [23,24,25]. These complexes are subsequently displayed on the cell surface, where they are recognized by CD8^+^ T cells to elicit a cellular immune response [26]. Immune escape mechanisms within the tumor microenvironment (TME) can substantially compromise the antitumor immune efficacy attributable to PSMB9. Although PSMB9 enhances the antigen processing and presentation capacity of CRC cells, CRC often resides within an immunosuppressive TME. This milieu is typically characterized by dense infiltration of M2 macrophages, regulatory T cells (Tregs), and myeloid-derived suppressor cells (MDSCs), accompanied by upregulated expression of immune checkpoint molecules such as PD-L1, while effector T cells are frequently in a state of functional exhaustion [27,28]. Even when antigen presentation is successfully accomplished on the tumor cell surface, it tends to fail in effectively initiating and activating antitumor immune responses, thereby largely diminishing the potential tumor-suppressive effects of PSMB9 mediated by antigen presentation. Meanwhile, the role of PSMB9 in regulating pathways associated with the intrinsic malignant progression of tumor cells appears to be less affected by the TME, allowing it to potentially sustain the malignant evolution of CRC.

The catalytic β1i subunit of the immunoproteasome is encoded by the low molecular weight protein 2 (LMP2)/PSMB9 gene [8,29]. Previous studies have demonstrated that high expression of LMP2 can not only promote the formation of tumor masses in the early stage of primary tumorigenesis, but also further drive tumor growth and malignant progression [30,31]. Through a series of *in vitro* functional experiments and mechanistic investigations, this study confirmed that PSMB9 exerts a significant oncogenic function during the occurrence and development of CRC. This conclusion is in stark contrast to the previously reported tumor-suppressive role of PSMB9 in cervical cancer [32]. Such functional divergence is likely driven by cell of origin differences, microenvironmental cues, and distinct signaling networks across tumor types. CRC arises from intestinal epithelial cells within a microenvironment enriched in gut microbiota metabolites, inflammatory cytokines, and Wnt/β-catenin pathway hyperactivation. In contrast, cervical cancer originates from squamous or glandular epithelium, with oncogenesis tightly linked to human papillomavirus (HPV) infection, estrogen signaling, and unique immune infiltration profiles. This tissue-specific context dictates the molecular function of PSMB9, highlighting that its biological role is not intrinsic but highly context-dependent. Further mechanistic studies are warranted to dissect the precise regulatory networks governing PSMB9′s dual roles in cancer.

Our findings provide new insights into the complex role of PSMB9 in CRC. Previous studies have pointed out that Gankyrin can downregulate PSMB9 expression via the tumor necrosis factor (TNF) signaling pathway, thereby affecting CRC progression and metastasis [33]. Some studies have also reported differential PSMB9 expression in early-onset CRC, but its specific function and mechanism remain unclear [34]. In this context, the present study not only experimentally confirms that PSMB9 exerts a pro-tumor effect in CRC, but also reveals that it regulates tumor malignant behaviors by activating the PI3K/Akt signaling pathway. This thus clarifies the key role of PSMB9 in CRC oncogenesis and development, and provides a theoretical basis for subsequent targeted intervention strategies.

Nevertheless, this study has several limitations. First, our findings rely primarily on *in vitro* experiments and lack *in vivo* animal model validation and pharmacologically treated models. Second, the regulatory crosstalk between PSMB9 in tumor cells and the tumor immune microenvironment also merits further in-depth investigation. In light of these limitations, future studies will focus on systematically evaluating the therapeutic potential of PSMB9-targeted intervention in combination regimens for CRC using preclinical animal models, and will simultaneously explore the underlying immunomodulatory mechanisms to clarify how PSMB9 shapes the tumor immune microenvironment.

## 5 Conclusion

In summary, our study demonstrates that PSMB9 promotes the malignant progression of CRC cells by activating the PI3K/Akt signaling pathway. These findings not only clarify the pro-tumor role and molecular mechanism of PSMB9 in CRC, but also identify PSMB9 as a potential novel therapeutic target, thereby providing a promising direction for the development of clinical treatment strategies for this disease.

## Data Availability

This study used the following publicly available databases and online analysis tools: TCGA (https://portal.gdc.cancer.gov/), GEO (dataset GSE39582, https://www.ncbi.nlm.nih.gov/geo/), GEPIA (http://gepia.cancer-pku.cn/), and HPA (https://www.proteinatlas.org/). The original experimental data generated during this study are available from the Corresponding Author upon reasonable request.
